# Physical Fitness Promotion among Adolescents: Effects of a Jump Rope-Based Physical Activity Afterschool Program

**DOI:** 10.3390/children7080095

**Published:** 2020-08-14

**Authors:** Xiaofeng Yang, Joonyoung Lee, Xiangli Gu, Xiaoxia Zhang, Tao Zhang

**Affiliations:** 1 School of Leisure Sport, Shanghai University of Sport, Shanghai 200438, China; yangxiaofeng5940@sina.com.cn; 2 Department of Kinesiology, Health Promotion and Recreation, University of North Texas, Denton, TX 76203, USA; 3 Department of Kinesiology, University of Texas at Arlington, Arlington, TX 76019, USA; Xiangli.Gu@uta.edu (X.G.); xiaoxia.zhang@mavs.uta.edu (X.Z.)

**Keywords:** physical activity, physical fitness, jump rope, afterschool program, Chinese adolescents, health promotion

## Abstract

The major purpose of this study was to examine the effects of a jump rope-based physical activity afterschool program on middle school students’ physical fitness. Sixty students (*M*_age_ = 13.37, *SD* = 0.58; 53.3% female) participated in a 12-week jump rope-based afterschool program (45 min/time, three times/week). Participants were randomly assigned to three groups: (a) freestyle rope skipping (*N* = 20), traditional jump rope (*N* = 20), and a control group (*N* = 20). Physical fitness tests, including muscular strength (standing long jump, right-hand grip, and left-hand grip), flexibility, body composition, and bone mineral density (BMD) were measured in pre- and post-tests. A 2 (time) × 3 (groups) repeated measure multivariate analysis of variance (MANOVA) was performed. The results found significant improvements in muscular strength (standing long jump, right-hand grip, and left-hand grip) in both intervention groups (*p* < 0.001; *d*s = 0.2–0.44). Only the freestyle rope skipping group had increased BMD (*p* < 0.05, *d* = 0.33). Compared to the traditional jump rope, the freestyle rope skipping group showed significantly higher improvement in flexibility (*p* < 0.05, *d* = 0.83). These findings suggest that the jump rope-based afterschool program with freestyle rope skipping would be more effective than traditional jump rope to promote physical fitness performance among adolescents.

## 1. Introduction

According to recent global physical activity (PA) guidelines, adolescents should engage in at least one hour of moderate to vigorous physical activity (MVPA) each day to gain sufficient health benefits [1,2,3,4]. However, more than 80% of adolescents failed to meet the daily MVPA recommendations worldwide [5], and only 29.9% of Chinese adolescents engage in the recommended daily MVPA. Adolescence (11–15 years old) is a crucial developmental stage for physiological maturation and physical fitness development [6]. Nevertheless, over one-third of adolescents are either overweight or obese [1,7,8,9], and a substantial decline in cardiovascular and muscular fitness in adolescents has been reported across the globe [10,11,12,13]. Low levels of physical fitness during adolescence tend to track into adulthood and lead to both short- and long-terms health risks and chronic disease [2,14,15,16]. It is an urgent need to implement effective interventions aiming to promote adolescents’ PA participation and improve their physical fitness. 

Jump rope-based PA intervention is suggested to promote adolescents’ PA participation and overall physical fitness within and beyond the school setting [17,18,19]. Jump rope is highly accessible, enjoyable, and affordable to students [20,21]. Jump rope exercise is a whole-body movement (upper and lower body) that requires rhythm, coordination, agility, speed, and strength [19,22]. For example, when adolescents start jump rope exercise, their arms need to keep rotating, and their bodies should maintain and coordinate on repetitive vertical jumps with rhythm [23]. 

A burgeoning amount of research has started to involve jump rope in physical education (PE) and sport training programs and has observed favorable physical (i.e., PA, cardiovascular fitness, coordination, and jump skills) and psychological outcomes (i.e., self-confidence and peer relationships) [23,24,25,26]. For instance, Partavi [25] implemented jump rope intervention in secondary PE sessions (15 min/four consecutive PE lessons), and they found significantly increased MVPA during PE classes and cardiovascular fitness. After inserting jump rope intervention into warm-up routines (10–20 min) for 8–10 weeks, Trecroci et al. [23] found positive changes in balance and motor coordination among preadolescent soccer athletes. Most recently, García-Pinillos and colleagues [26] also demonstrated the significant impact of jump rope on improvements in physical fitness and running performance among amateur runners in a 10-week intervention. Although the effects of jump rope on improving physical fitness levels have been proven in settings such as PE or sports, limited research has implemented jump rope in afterschool programs. 

Afterschool programs can be efficacious for implementing jump rope exercise since they allow students to participate in a variety of movements and physical activities [27,28]. Previous evidence has shown that an afterschool PA program brings multiple health benefits, including increased PA behaviors [29], working memory [30], motor skills competence [31], reduced body fat [32], and increased academic motivation and effort [33]. It was also noted that adolescents’ MVPA was strongly associated with supervised PA and accessibility to sports equipment and facilities during afterschool programs [34]. Because jump rope exercise is relatively less affected by space and location compared to other physical activities (e.g., basketball, soccer, football), jump rope-based PA afterschool programs could be considered a sustainable activity to promote adolescents’ physical fitness. 

Jump rope is an ever-evolving sport skill, which can be constantly developed and improved. The traditional jump rope style, the most common rope exercise, focuses on frequency in a limited time, in which players need to make as many jumps as possible. In recent years, freestyle rope skipping, as a new style of jump rope exercise, has been introduced to students in schools. Freestyle rope skipping emphasizes the variations and interactions between rope swings and jumps, in which players may modify the rope swing periodically during the time limit [35]. In addition, freestyle rope skipping is more flexible and creative, as it involves different elements, such as music, dance, martial arts, and other performances. Both jump rope styles (i.e., traditional jump rope and freestyle rope skipping) have evolved from recreational games into official competitions and have become one of the most popular physical activities in public secondary schools in China [36]. However, to our knowledge, no research has compared the effects of different jump rope styles (i.e., traditional jump rope vs. freestyle rope skipping) on adolescents’ physical fitness levels. Therefore, the primary purpose of this study was two-fold: (1) to examine the effects of a 12-week jump rope-based afterschool program on adolescents’ physical fitness; (2) to examine whether the intervention effects differ based on different styles of jump rope exercise (freestyle rope skipping vs. traditional jump rope) among adolescents. We hypothesized that a jump rope-based afterschool program would be effective in improving adolescents’ physical fitness, and the freestyle rope skipping style would be more effective in improving physical fitness than the traditional jump rope style. 

## 2. Materials and Methods

### 2.1. Participants and Study Design

Participants were 60 middle school students (*M*_age_ = 13.37, *SD* = 0.58; 53.3% female) recruited from a public middle school in Shanghai, China. The inclusion criteria for participant recruitment were (a) students who returned the parental consent and assent forms, (b) students with no physical or psychological disabilities, and (c) no student athletes. The researchers received the university’s ethical approval according to the Declaration of Helsinki (Project identification code: SU-1116) prior to undertaking the data collection. Among approximately 1800 students enrolled in 24 classrooms (i.e., about 75 students in each class; grades 6–8) in the middle school, 60 students were recruited according to the estimated sample size (*N* = 51; f2 = 0.35, π = 0.80, α = 0.05) from G*power 3.1 [37]. 

### 2.2. Procedure

The data collection and intervention were administrated during the afterschool program period (5:00 p.m.–6:00 p.m.). The Shanghai middle school system commonly provides middle school students with an afterschool program and unstructured free play (i.e., self-directed play without specific learning objectives) before dinner (6:00 p.m.–7:00 p.m.) and evening class sessions (7:00 p.m.–9:00 p.m.). Participants were recruited via fliers introducing the 12-week jump rope intervention project. Sixty students (*N* = 41 (6th grade), *N* = 16 (7th grade), and *N* = 3 (8th grade)) directly contacted the principal investigator and conveyed their interest and willingness to participate in the study. Parental consent and student assent forms were obtained before undertaking the project. 

**Figure 1:**
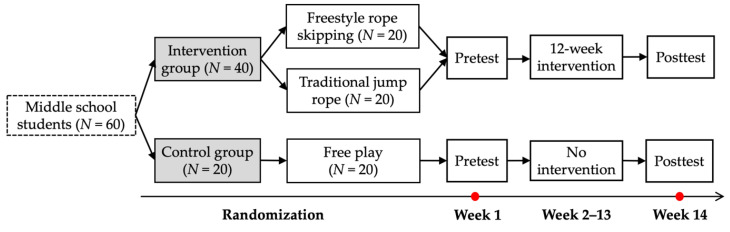
Procedures of the summarized intervention study.

Figure 1 presents the procedures of this intervention study. By applying random numbers generated from Microsoft Excel 2016, the 60 students were randomly assigned into three groups: freestyle rope skipping (*N* = 20; 12 girls), traditional jump rope (*N* = 20; 10 girls), and control (*N* = 20; 10 girls) groups. Pre- (week 1) and post-tests (week 14) were conducted in the indoor gym in the middle school. Prior to the measurements, trained graduate assistants instructed and demonstrated each physical fitness assessment to assure student comprehension. The intervention was administrated during the afterschool program for 12 weeks (weeks 2–13 in Figure 1). The participants from the control group engaged in free play without specific PA instructions.

### 2.3. Intervention Program

A summary of the intervention program is displayed in Table 1. Students from the freestyle rope skipping group and the traditional jump rope group participated in the 45-min (5:00 p.m.–5:45 p.m.) jump rope-based PA afterschool program guided by two jump rope instructors, respectively. This intervention was undertaken three times (Monday, Wednesday, and Friday) a week for 12 weeks (i.e., 36 sessions = three sessions × 12 weeks) in the indoor gym in the middle school. The 45-min jump rope-based PA afterschool program included: (a) 10 min of warm-up and instruction, (b) 30 min of jump rope-based exercise, and (c) 5 min of cooldown and stretching. During the warm-up and instruction, students were instructed to jog around the gym and stretch for warm-up. After that, instructors delivered skill-based lessons (i.e., explanation and demonstration) and set up goals (e.g., frequency, time, intensity) to motivate students’ engagement in the jump rope activity. For example, the participants set goals about how many jumps without stopping they could do (frequency) within a certain time (time) and achieving specified pedometer step counts (intensity). Bi-weekly lesson plans (i.e., repeating the same skills for two weeks) were applied to the 30 min of jump-rope activities to provide enough time for students to acquire essential skills in each lesson. During the 5 min of cooldown and stretching, students engaged in stretching exercises to allow the recovery of heart rate and breathing.

In the freestyle rope skipping group, team performance and sharing fun with peers were emphasized. The primary instructor taught the formation of team performance and rhythm practice with music. Freestyle rope skipping allowed participants to engage in various rope skipping techniques both individually and as part of a team performance. In contrast, the traditional jump rope group focused on individual frequency and speed. The students performed the jump rope exercises based on one of the instructor’s signals and recorded the number of jumps they did in the exercise log. The control group engaged in self-directed unsupervised free play. According to short interviews with students from the control group, they walked around on campus during the afterschool program.

As a fidelity check of the intervention, the instructors checked the students’ afterschool program attendance; 97% were present for the intervention. Stopwatches were used to check the afterschool program duration. Digital pedometers (model: HS800822, B&K Electronic Technology Co., Ltd., Guangdong, China) were also employed to ensure the PA intensity of the intervention. Scruggs [38] suggested a cut point interval of 82–88 steps/min for middle school students’ MVPA in PE. Therefore, we set up step count goals (2460–2640 steps/30 min) to maintain the intensity of intervention in the program. 

The primary afterschool program instructor (40 years old) is an associate professor in PE and health promotion with a specialization in jump rope and dragon boat majors and has rich experience in teaching PA classes at a leading university in Shanghai, China. Another afterschool program instructor (24 years old) was a graduate teaching assistant enrolled in the M.S. program in PE with a concentration in leisure sport and jump rope. The program instructors’ coaching experience of jump rope was 3 to 18 years at the K-12 level and 2 to 10 years at the undergraduate level. The instructors’ major roles were to (1) teach essential rope skipping skills with explanation and demonstration; (2) organize the skill practice into a series of developmentally appropriate blocks; and (3) provide individualized suggestions and feedback about skills for students upon request.

### 2.4. Measures

#### 2.4.1. Muscular Strength

Students’ muscular strength was assessed using standing long jump and hand grip. In the standing long jump test, students stood on a line marked on the ground. They placed their feet slightly apart, swinging the arms and bending the knees, and then jumped as far as possible. The students were required to jump three times, and the longest jump distance was recorded and utilized in the data analysis. Students’ handgrip strength was evaluated using the Camry digital handgrip dynamometer (model: EH101, Camry Industries Company Ltd., Kowloon, Hong Kong, China). Both left- and right-hands grip strength (kg) were measured three times in a standing position, and the highest number was used for the final data analyses in this study. Previous research showed that the standing long jump and handgrip tests were highly reliable and valid for measuring muscular strength [39]. 

#### 2.4.2. Flexibility 

The toe-touch test was used to measure students’ lower body flexibility. The students stood on a box (5.75 × 2.5 × 4.4″) bare-footed and maintained straight arms and knees during testing and bent over to try to touch the box for 10 s. The research assistants measured the distance (cm) between each students’ fingers and the box using a measuring tape if participants could not reach it. If the students’ fingertips did not contact the box, the scores were negative; if the student reached it, the scores were recorded as positive. The test’s reliability and validity were shown in previous research [40]. 

#### 2.4.3. Body Composition

Students’ body composition was assessed using waist circumference. Research assistants measured students’ bare waist circumference at a standing posture using a measuring tape. The research assistants started at the top of the participant’s hip bone at the belly button level and wrapped the tape around the waist. The measurement was performed twice, and the mean score (cm) was recorded in this study. 

#### 2.4.4. Bone Mineral Density 

Bone mineral density (BMD) from the students’ right heel was assessed using the Sahara clinical bone sonometer (Hologic, MA, USA). This equipment estimated the students’ BMD in g/cm^2^ by measuring the transmission of high-frequency sound waves through the heel. Students were instructed to sit on the chair, and an oil-based coupling gel was applied to their heel. A previous study showed the validity and reliability of the equipment [41].

### 2.5. Data Analysis

Data were screened prior to the data analysis and no missing data, outliers, or normality issues were found in the present study. Three steps were used to analyze the data. First, one-way analysis of variance (ANOVA) was performed to test the differences in baseline physical fitness variables among groups (freestyle rope skipping vs. traditional jump rope vs. control group). Second, a 2 (time (pre- and post-test)) × 3 (groups) repeated measures multivariate analysis of variance (MANOVA) was conducted to examine the intervention effects and group effects on physical fitness variables. Finally, a further univariate ANOVA and post hoc tests using Bonferroni adjustment were performed to explore group differences. The effect size for the eta squared (η^2^) was identified as >0.01 (small), ≥0.06 (medium), and ≥0.14 (large) [42]. To show statistical differences between the pre-test and post-test study variables by each group, we calculated the effect size using Cohen’s *d* with the following criteria: ≥0.20 (small), ≥0.50 (medium), and ≥0.80 (large) [43]. An alpha level of 0.05 was used for all data analyses.

## 3. Results

### 3.1. Baseline Descriptive Statistics by Groups 

Table 2 displays the descriptive results of the study variables in the pre-test by the three groups. The one-way ANOVA results showed no significant group differences in study variables (*p* > 0.05) in the pre-test (before the intervention).

### 3.2. Effects of the Jump Rope-Based PA Afterschool Program

Table 3 presents the changes in study variables across the pre-test and post-test, stratified by groups. The repeated measures MANOVA demonstrated a statistically significant time by group interaction effect (Wilks’s lambda = 0.15, F (10, 106) = 16.19, *p* < 0.001, η^2^ = 0.60). The follow-up univariate ANOVA test indicated that the three groups’ improvements significantly differed over time in standing long jump (F (2, 57) = 7.60, *p* < 0.001, η^2^ = 0.21), right-hand grip (F (2, 57) = 31.16, *p* < 0.001, η^2^ = 0.52), left-hand grip (F (2, 57) = 67.85, *p* < 0.001, η^2^ = 0.71), flexibility (F (2, 57) = 5.42, *p* < 0.01, η^2^ = 0.16), and body composition (F (2, 57) = 4.33, *p* < 0.05, η^2^ = 0.13), but not in BMD (F (2, 57) = 2.19, *p* > 0.05, η^2^ = 0.07). The Bonferroni post hoc analysis demonstrated a significant improvement of muscular strength (standing long jump, right-hand grip, and left-hand grip), with a small effect size (*p* < 0.001; *ds* = 0.2–0.44), in both intervention groups (freestyle rope skipping and traditional jump rope) compared to the control group. Notably, the freestyle rope skipping group significantly improved in flexibility, with a medium effect size (*p* < 0.05; *d* = 0.55) compared to the traditional jump rope and control groups, while body composition increased significantly in the traditional jump rope group compared with the control group (*p* < 0.05, *d* = 0.77). Although there was no group difference, the freestyle rope skipping group had increased BMD with a small effect size (*p* < 0.05; *d* = 0.33) across time.

### 3.3. Freestyle Rope Skipping vs. Traditional Jump Rope 

**Figure 2:**
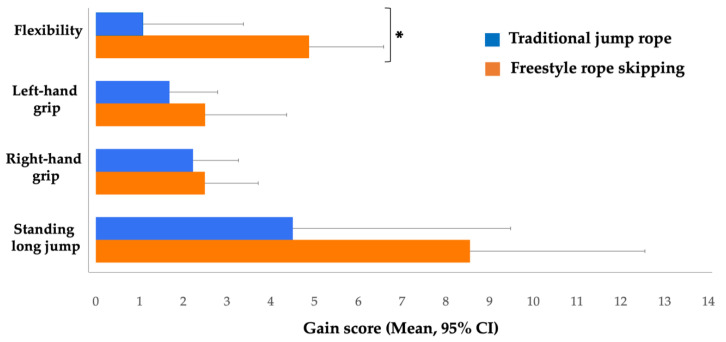
Gain in scores of physical fitness performances showing group differences between freestyle rope skipping and traditional jump rope. The asterisk represents a significant group difference between intervention groups, * *p* < 0.05.

The univariate ANOVA analysis testing group differences between freestyle rope skipping and traditional jump rope showed no significant differences (*p* > 0.05) in muscular strength (standing long jump, right-hand grip, and left-hand grip), while effect size (Cohen’s *d*) analysis between the two groups indicated that freestyle rope skipping was more effective in improving students’ standing long jump (*d* = 0.40) and left-hand grip (*d* = 0.23) than traditional jump rope. Furthermore, the flexibility score was statistically higher in the freestyle rope skipping group compared to the traditional jump rope group (*p* < 0.05, *d* = 0.83) (see Figure 2).

## 4. Discussion

The major purpose of this study was to examine the effects of a 12-week jump rope-based PA afterschool intervention (i.e., freestyle rope skipping and traditional jump rope) on middle school students’ physical fitness performances. The results of this study indicated significant improvements over time of physical fitness performances (standing long jump, right-hand grip, left-hand grip, and flexibility) in both intervention groups. Only the freestyle rope skipping group increased in BMD. However, the control group did not show any significant betterment in physical fitness performances in the post-test. The comparison between freestyle rope skipping and traditional jump rope indicated more improvements in muscular strength and flexibility in the freestyle rope skipping group. These results added empirical evidence to the effectiveness on physical fitness of a jump rope-based PA afterschool program, especially the effectiveness on muscular strength and flexibility of the freestyle rope skipping intervention. 

After students participated in the 12-week jump rope-based PA afterschool program, statistically significant improvements in standing long jump were demonstrated in the group of freestyle rope skipping (5.29%, *d* = 0.40) and traditional jump rope (2.79%, *d* = 0.20). Yet, students in the control group showed deterioration in the standing long jump test (−2.14%, *d* = 0.11). This finding also supports previous literature that suggests that jump-based PA intervention can enhance adolescents’ standing long jump performance [39]. Interestingly, Colakoglu et al. [39] applied heavy jump ropes (weight: 1.3–1.5 lb) to the weighted jump rope group to compare the effects of 15 min of jump rope training with other groups (standard jump rope group and control group), and found a significant increase in standing long jump among the female student athletes from the weighted jump rope group. Although our study used standard jump ropes (weight: 0.2–0.4 lb) for both intervention groups, the intervention groups’ performance in the standing long jump was significantly improved. This may be due to the differences in duration and intensity from the previous study. We implemented 30 min of jump rope practice time and MVPA levels of intensity. In future studies, it would be interesting to examine differences in the effects of the jump rope-based PA intervention by implementing different weights of jump ropes for intervention groups in a school setting. Additionally, it is worth noting that the freestyle rope skipping group’s standing long jump performance showed more improvement than that of the traditional jump rope group. This difference might be attributed to the fact that freestyle rope skipping has higher requirements for various jumps (i.e., short and long) in different directions (i.e., front, back, right, and left) [19,44]. Furthermore, the different skipping styles in freestyle rope skipping also require the integration of physical agility and explosive strength [45]. Thus, it will be an effective strategy to implement the freestyle rope skipping PA program for adolescents to enhance their muscular strength.

Consistent with previous study [39], jump rope-based intervention groups (freestyle rope skipping and traditional jump rope) demonstrated significant improvement in hand grip strength compared to the control group. Specifically, students in the freestyle rope skipping group considerably enhanced their hand grip strength (right hand: 12.28%, *d* = 0.41; left hand: 13.38%, *d* = 0.42), and the traditional jump rope group’s students also notably increased their hand grip strength (right hand: 11.41%, *d* = 0.44; left hand: 9.79%, *d* = 0.31). However, participants in the control group had significantly decreased in their grip strength of both hands (right hand: −26.32%, *d* = 0.88; left hand: −37.82%, *d* = 1.53). Compared to traditional jump rope, the freestyle rope skipping group demonstrated slightly more increased left-hand grip strength. This may be due to the variation of rotating jump rope skills using body strength (i.e., wheel, double Dutch), which demands the force of both arms in freestyle rope skipping [19]. Hand grip strength is a significant indicator of multiple health outcomes (e.g., BMI, muscular strength) [46,47] and is a foundation of skill performance (i.e., martial arts) [48]. Adolescents with greater hand grip strength were found to have greater self-concept [48]. 

Body flexibility is one critical component of physical fitness, and being flexible is associated with a lower risk of injuries and better performance in physical activities and daily life activities [49,50]. The 12-week jump rope-based PA afterschool intervention in the present study revealed a significant improvement of flexibility among adolescents. Although the control group seemed to show significant changes in flexibility (633.30%, *d* = 0.14), their flexibility was obviously lower than that of the other two intervention groups. The finding is aligned with a few previous studies among different groups of adolescents (males, intellectual disabilities) [51,52]. Compared to previous studies, one unique aspect of this study was that freestyle rope skipping was demonstrated to be more effective on flexibility (163.97%, *d* = 0.55) than traditional jump rope (20.00%, *d* = 0.19). This might have been due to the fact that freestyle rope skipping integrates various movements, such as hand rolls, flips, flying feet, and assorted steps, with the use of more muscles [19].

Waist circumference is an important indicator to estimate individual body fatness [53]. Waist circumference is a biomarker for obesity and some diseases such as diabetes, heart disease, arthritis, and cancer [54,55]. Interestingly, the waist circumference of the traditional jump rope group increased significantly (7.88%; *d* = 0.84) while other two groups had no significant changes. The findings contradicted previous studies’ results that jump rope-based interventions significantly reduced participants’ body fatness [25,39]. This might be due to different measures, in which body mass index (BMI) was frequently used in the previous research [25,39]. Although it might be difficult to elucidate this contradictive result, our study recognized that the participants’ waist circumferences were within the non-risky range of overweight or obese (≤88.9–101.6 cm) [56].

It was documented that various jump-based training (intensity, direction, and type) allowed for increasing body BMD [57]. To our surprise, there were no significant group differences or intervention effects for BMD. This might due to the limited intervention period in the present study. Kato et al. [58] demonstrated considerable changes in torso BMD among female college students after 6 months of jump training (i.e., two-legged maximum vertical jumps 10 times/three days/24 weeks). Given only a 12-week intervention in this study, freestyle rope skipping showed a small to medium effect size on BMD (7.14%, *d* = 0.33). This finding suggests that freestyle rope skipping would be more beneficial and effective in enhancing bone development among adolescents. A long-term jump rope-based intervention is warranted to test its effects on bone development among adolescents. 

The findings of this study provided empirical evidence of a jump rope-based PA afterschool program in promoting adolescents’ physical fitness performances, such as muscular strength and flexibility. Several limitations still existed in this study. First, this study did not objectively monitor the control group’s PA during the afterschool program, while a series of short interviews were processed with participants from the control group regarding their physical activities every Friday during the 12-week intervention. Second, this study did not measure psychological factors concerning the afterschool program. Freestyle rope skipping is regarded as more fun and exciting than traditional jump rope [24]. Future studies may consider examining participants’ changes in motivation or enjoyment with regard to different rope skipping interventions. Third, there were no follow-up tests (e.g., 3 months later) in the current study. At the end of the project, researchers provided jump ropes to students in all groups and encouraged them to maintain exercise using jump ropes. Thus, follow-up measures are suggested in the future to identify the lasting effects of the 12-week jump rope-based PA afterschool program on students’ physical fitness performances or healthy behavioral changes.

## 5. Conclusions

To our knowledge, this study is the first investigation to examine the effects of a 12-week jump rope-based PA intervention in an afterschool programs on adolescents’ physical fitness performances and, furthermore, it compared the effects between freestyle rope skipping and traditional rope jumping. Both intervention groups showed significant enhancements in muscular strength and flexibility after the 12-week jump rope-based PA intervention. The freestyle rope skipping group demonstrated better improvements in all study variables compared to the traditional rope jump group. PE teachers and afterschool program administrators are advised to adopt freestyle rope skipping to improve students’ physical fitness performances. A jump rope-based PA program is recommended as a feasible curriculum element to be embedded in afterschool programs. 

## Figures and Tables

**Table 1:** The summary of the intervention program.

Variables	Freestyle Rope Skipping	Traditional Jump Rope	Control
Style	Dance, gymnastic, fancy skip, martial arts	Bounce	Free play
Leaning objects	Team performance and enjoyment	Frequency and speed	N/A
Sessions (times/weeks)	36 sessions (three times/12 weeks)	36 sessions (three times/12 weeks)	12 weeks
Intensity (# steps)	2460–2640 steps (MVPA)	2460–2640 steps (MVPA)	N/A
Training time	45 min (10 min: warm-up and instruction ➔ 30 min: jump rope activities ➔ 5 min: cooldown and stretching)	45 min (10 min: warm-up and instruction ➔ 30 min: jump rope activities ➔ 5 min: cooldown and stretching)	N/A
Bi-weekly lesson plan			
Week 2–3	Single freestyle	Basic rope skills	N/A
Week 4–5	Long rope freestyle	Single rope speed	
Week 6–7	Chinese wheel freestyle	Double under speed	
Week 8–9	Double Dutch freestyle	Triple under speed	
Week 10–11	Single + Long rope freestyles	Basic rope skills + Single rope speed	
Week 12–13	Chinese wheel + Double Dutch freestyle	Double + Triple under speed	

Note. N/A = not applicable; # = number of participants’ steps for 30-min jump rope activities.

**Table 2:** Descriptive results of the study variables in the pre-test by the three groups (*N* = 60).

Variables	Freestyle RS (*N* = 20)	Traditional JR (*N* = 20)	Control (*N* = 20)	Group Difference
	*M ± SD*	*M ± SD*	*M ± SD*	*F*/*p*
Age	13.40 ± 0.59	13.55 ± 0.68	13.15 ± 0.36	2.54/0.08
Muscular strength (SLJ)	161.50 ± 20.96	161.25 ± 26.05	170.25 ± 32.36	0.72/0.48
Muscular strength (RHG)	20.27 ± 5.84	19.46 ± 4.69	21.24 ± 7.12	0.44/0.64
Muscular strength (LHG)	18.69 ± 6.35	19.12 ± 5.44	21.23 ± 5.47	1.10/0.33
Flexibility	2.97 ± 10.19	5.35 ± 4.26	−0.15 ± 6.97	2.67/0.07
Body composition (WC)	69.00 ± 7.45	62.80 ± 8.32	66.35 ± 8.92	2.83/0.06
BMD	0.45 ± 0.09	0.40 ± 0.08	0.43 ± 0.09	0.68/0.50

Note. Freestyle RS = freestyle rope skipping; Traditional JR = traditional jump rope; SLJ = standing long jump; RHG = right-hand grip; LHG = left-hand grip; sec = seconds; WC = waist circumference; BMD = bone mineral density; N/A = not applicable.

**Table 3:** Descriptive results of study variables between pre-test and post-test (*N* = 60).

Variables	Groups	Pre-test, *M* ± *SD*	Post-test, *M* ± *SD*	Post–Pre, *M* (%)	*p* (Group × Time)	*d*
Muscular strength (SLJ)	Freestyle RS	161.50 ± 20.96	170.05 ± 21.51	8.55 (5.29)	<0.001	0.40
	Traditional JP	161.25 ± 26.05	165.75 ± 21.78	4.50 (2.79)		0.20
	Control	170.25 ± 32.36	166.60 ± 32.57	−3.65 (−2.14)		0.11
Muscular strength (RHG)	Freestyle RS	20.27 ± 5.84	22.76 ± 6.27	2.49 (12.28)	<0.001	0.41
	Traditional JP	19.45 ± 4.69	21.67 ± 5.28	2.22 (11.41)		0.44
	Control	21.24 ± 7.12	15.65 ± 5.32	−5.59 (−26.32)		0.88
Muscular strength (LHG)	Freestyle RS	18.69 ± 6.35	21.20 ± 5.50	2.50 (13.38)	<0.001	0.42
	Traditional JP	19.12 ± 5.44	20.80 ± 5.36	1.68 (8.79)		0.31
	Control	21.23 ± 5.47	13.20 ± 5.01	−8.03 (−37.82)		1.53
Flexibility	Freestyle RS	2.97 ± 10.19	7.85 ± 7.12	4.87 (163.97)	<0.01	0.55
	Traditional JP	5.35 ± 4.26	6.42 ± 6.59	1.07 (20.00)		0.19
	Control	−0.15 ± 6.97	0.80 ± 5.72	0.95 (633.30)		0.14
Body composition (WC)	Freestyle RS	69.00 ± 7.45	68.93 ± 6.05	−0.07 (−0.10)	<0.05	0.01
	Traditional JP	62.80 ± 8.32	67.75 ± 7.51	4.95 (7.88)		0.84
	Control	66.35 ± 8.92	64.25 ± 13.39	−2.10 (−3.17)		0.18
BMD	Freestyle RS	0.42 ± 0.09	0.45 ± 0.09	0.03 (7.14)	>0.05	0.33
	Traditional JP	0.40 ± 0.08	0.40 ± 0.08	0.00 (0.00)		0.00
	Control	0.43 ± 0.09	0.42 ± 0.08	−0.01 (−2.33)		0.11

Note. Each group included twenty participants; *d =* Cohen’s *d;* Freestyle RS = freestyle rope skipping; Traditional JR = traditional jump rope; SLJ = standing long jump; RHG = right-hand grip; LHG = left-hand grip; WC = waist circumference; BMD = bone mineral density.

## References

[1] Piercy K.L., Troiano R.P., Ballard R.M., Carlson S.A., Fulton J.E., Galuska D.A., George S.M., Olson R.D. (2018). The Physical Activity Guidelines for Americans. J. Am. Med. Assoc..

[2] United States Department of Health and Human Services Physical Activity Guidelines for Americans. https://health.gov/paguidelines/second-edition/.

[3] Tremblay M.S., Carson V., Chaput J.-P., Connor Gorber S., Dinh T., Duggan M., Faulkner G., Gray C.E., Gruber R., Janson K. (2016). Canadian 24-Hour Movement Guidelines for Children and Youth: An Integration of Physical Activity, Sedentary Behaviour, and Sleep. Appl. Physiol. Nutr. Metab..

[4] The Australian Government Department of Health (2019). Australian 24-Hour Movement Guidelines for Children and Young People (5 to 17 Years): An Integration of Physical Activity, Sedentary Behaviour, and Sleep.

[5] Guthold R., Stevens G.A., Riley L.M., Bull F.C. (2020). Global Trends in Insufficient Physical Activity among Adolescents: A Pooled Analysis of 298 Population-Based Surveys with 1·6 Million Participants. Lancet Child. Adolesc. Health..

[6] Haywood K.M., Getchell N. (2019). Life Span. Motor Development.

[7] Rezende L.F.M., Lopes M.R., Rey-Lopez J.P., Matsudo V.K.R., Luiz O.D.C. (2014). Sedentary Behavior and Health Outcomes: An Overview of Systematic Reviews. PLoS ONE.

[8] Tremblay M.S., LeBlanc A.G., Kho M.E., Saunders T.J., Larouche R., Colley R.C., Goldfield G., Connor Gorber S. (2011). Systematic Review of Sedentary Behaviour and Health Indicators in School-Aged Children and Youth. Int. J. Behav. Nurt. Phys. Act..

[9] Chinapaw M.J.M., Proper K.I., Brug J., van Mechelen W., Singh A.S. (2011). Relationship between Young Peoples’ Sedentary Behaviour and Biomedical Health Indicators: A Systematic Review of Prospective Studies. Obes. Rev..

[10] Zhu Z., Yang Y., Kong Z., Zhang Y., Zhuang J. (2017). Prevalence of Physical Fitness in Chinese School-Aged Children: Findings from the 2016 Physical Activity and Fitness in China—The Youth Study. J. Sport Health Sci..

[11] Cohen D., Voss C., Taylor M., Delextrat A., Ogunleye A., Sandercock G. (2011). Ten-Year Secular Changes in Muscular Fitness in English Children. Acta Paediatr..

[12] Müllerová D., Langmajerová J., Sedláček P., Dvořáková J., Hirschner T., Weber Z., Müller L., Brázdová Z.D. (2015). Dramatic Decrease in Muscular Fitness in Czech Schoolchildren over the Last 20 Years. Cent. Eur. J. Public Health.

[13] Tomkinson G.R., Lang J.J., Tremblay M.S. (2019). Temporal Trends in the Cardiorespiratory Fitness of Children and Adolescents Representing 19 High-Income and Upper Middle-Income Countries between 1981 and 2014. Br. J. Sports Med..

[14] Gu X., Zhang T., Chu T.L., Keller M.J., Zhang X. (2019). The Direct and Indirect Effects of Motor Competence on Adolescents’ Mental Health through Health-Related Physical Fitness. J. Sports Sci..

[15] Xiang M., Gu X., Jackson A., Zhang T., Wang X., Guo Q. (2017). Understanding Adolescents’ Mental Health and Academic Achievement: Does Physical Fitness Matter?. Sch. Psychol. Int..

[16] Gu X., Solmon M.A., Zhang T. (2012). Using Expectancy-Value Model to Examine Students’ Physical Activity Engagement and Cardiovascular Fitness in Physical Education. J. Sport Psychol..

[17] SHAPE America (2014). National Standards & Grade-Level Outcomes for K-12 Physical Education.

[18] Vidya K., Mullur M., Jyoti D.M. (2019). The Impact of Jump Rope Exercises on the Stress of 12 to 16 Years School Children. Int. J. Sports Physiol. Nutr. Phys. Educ..

[19] Cooper K. Benefits of Rope Jumping. http://www.jumpropeinstitute.com/.

[20] Ha A.S., Lonsdale C., Ng J.Y.Y., Lubans D.R. (2014). A School-Based Rope Skipping Intervention for Adolescents in Hong Kong: Protocol of a Matched-Pair Cluster Randomized Controlled Trial. BMC Public Health.

[21] Ha A.S., Burnett A., Sum R., Medic N., Ng J.Y.Y. (2015). Outcomes of the Rope Skipping “STAR” Programme for Schoolchildren. J. Hum. Kinet..

[22] Eler N., Acar H. (2018). The Effects of the Rope Jump Training Program in Physical Education Lessons on Strength, Speed and VO_2_ Max in Children. Univers. J. Educ. Res..

[23] Trecroci A., Cavaggioni L., Caccia R., Alberti G. (2015). Jump Rope Training: Balance and Motor Coordination in Preadolescent Soccer Players. J. Sport. Sci. Med..

[24] Ha A., Chan C., Sum R.K.W., Ng J.Y. (2014). Promoting Rope Skipping at Hong Kong Schools with Low and Mid Socioeconomic Statuses: An Ecological Perspective. Int. Rev. Soc. Sci..

[25] Partavi S. (2013). Effects of 7 Weeks of Rope-Jump Training on Cardiovascular Endurance, Speed, and Agility in Middle School Student Boys. Sport Sci..

[26] García-Pinillos F., Lago-Fuentes C., Latorre-Román P.A., Pantoja-Vallejo A., Ramirez-Campillo R. (2020). Jump-Rope Training: Improved 3-Km Time-Trial Performance in Endurance Runners via Enhanced Lower-Limb Reactivity and Foot-Arch Stiffness. Int. J. Sports Physiol. Perform..

[27] Kremer K.P., Maynard B.R., Polanin J.R., Vaughn M.G., Sarteschi C.M. (2014). Effects of After-School Programs with at-Risk Youth on Attendance and Externalizing Behaviors: A Systematic Review and Meta-Analysis. J. Youth Adolesc..

[28] Lee J., Zhang T., Chu T.L., Zhang X., Weiller-abels K.H., Keller J. (2019). Comprehensive School Physical Activity Program and Physical Literacy: Exploring Preservice Elementary Classroom Teachers’ Perspectives. Adv. Phys. Educ..

[29] Lee J., Zhang T., Chu T.L., Gu X., Zhu P. (2020). Effects of a Fundamental Motor Skill-Based Afterschool Program on Children’s Physical and Cognitive Health Outcomes. Int. J. Environ. Res. Public Health.

[30] Kamijo K., Pontifex M.B., O’Leary K.C., Scudder M.R., Wu C.T., Castelli D.M., Hillman C.H. (2011). The Effects of an Afterschool Physical Activity Program on Working Memory in Preadolescent Children. Dev. Sci..

[31] Lee J., Zhang T., Chu T.L., Gu X. (2020). Effects of a Need-Supportive Motor Skill Intervention on Children’s Motor Skill Competence and Physical Activity. Children.

[32] Martinez-Vizcaino V., Aguilar F.S., Gutiérrez R.F., Martínez M.S., Sánchez-López M., Martínez S.S., García E.L., Artalejo F.R. (2007). Assessment of an after-school physical activity program to prevent obesity among 9- to 10-year-old children: A cluster randomized trial. Int. J. Obes..

[33] Gatz J., Kelly A.M. (2018). Afterschool School Triathlon Training for 11- to 14-Year Old Girls: Influences on Academic Motivation and Achievement. Health Educ. J..

[34] Thornton C.M., Cain K.L., Conway T.L., Kerr J., Saelens B.E., Frank L.D., Sallis J.F. (2017). Relation of Adolescents’ Physical Activity to after-School Recreation Environment. J. Phys. Act. Health..

[35] Guo M., Li S. (2020). Construction of the Freestyle Rope Skipping Course Pattern from the Perspective of Sports Education Mode. Contemp. Sport. Technol..

[36] Hu G., Feng B., Fan G. (2020). Research on the Freestyle Rope Skipping Teaching to Promote the Middle School Youth Physical Health Development. Contemp. Sport. Technol..

[37] Faul F., Erdfelder E., Buchner A., Lang A.G. (2009). Statistical Power Analyses Using G*Power 3.1: Tests for Correlation and Regression Analyses. Behav. Res. Methods.

[38] Scruggs P.W. (2007). Middle School Physical Education Physical Activity Quantification: A Pedometer Steps/Min Guideline. Res. Q. Exerc. Sport.

[39] Colakoglu F., Karacan S., Guzel N., Baltaci G. (2017). Does the Weighted Rope Jump Training Affect the Motor Skills in Adolescent Female Volleyball Players?. Arch. Sport. Med. Physiother..

[40] González-Gálvez N., Poyatos M.C., Pardo P.J.M., Vale R.G.S., Feito Y. (2015). Effects of a Pilates School Program on Hamstrings Flexibility of Adolescents. Rev. Bras. Med. Do Esporte.

[41] Slatkovska L., Beyene J., Alibhai S.M.H., Wong Q., Sohail Q.Z., Cheung A.M. (2014). Effect of Whole-Body Vibration on Calcaneal Quantitative Ultrasound Measurements in Postmenopausal Women: A Randomized Controlled Trial. Calcif. Tissue Int..

[42] Lakens D. (2013). Calculating and Reporting Effect Sizes to Facilitate Cumulative Science: A Practical Primer for t-Tests and ANOVAs. Front. Psychol..

[43] Cohen J. (1988). Statistical Power Analysis for the Behavioral Sciences.

[44] Michiels Hernandez B.L., Gober D., Boatwright D., Strickland G. (2009). Jump Rope Skills for Fun and Fitness in Grades K-12. Joperd J. Phys. Educ. Recreat. Danc..

[45] Graham J.F., Brown L.E., Ferrigno V.A., Santana J.C. (2000). Training for Speed, Agility, and Quickness.

[46] Bohannon R.W. (2015). Muscle Strength: Clinical and Prognostic Value of Hand-Grip Dynamometry. Curr. Opin. Clin. Nutr. Metab. Care.

[47] Unal M., Kose O., Arik H., Guler F., Acar B., Yuksel H. (2018). Hand Grip Strength: Age and Gender Stratified Normative Data in Anatolian Population. Hand Microsurg..

[48] García P.L.R., Marcos L.T., Guillamón A.R., García-Cantó E., Pérez-Soto J.J., Casas A.G., Lopez P.T. (2014). Physical Fitness Level and Its Relationship with Self-Concept in School Children. Psychology.

[49] Mayo Clinic Staff Stretching: Focus on Flexibility. https://www.mayoclinic.org/healthy-lifestyle/fitness/in-depth/stretching/art-20047931.

[50] American Council on Exercise Flexibility Benefits. https://www.acefitness.org/education-and-resources/lifestyle/blog/6646/benefits-of-flexibility/.

[51] Chen C.C., Lin Y.C. (2012). Jumping Rope Intervention on Health-Related Physical Fitness in Students with Intellectual Impairment. J. Hum. Resour. Adult Learn..

[52] Arazi H., Jalali-Fard A., Abdinejad H. (2016). A Comparison of Two Aerobic Training Methods (Running vs Rope Jumping) on Health-Related Physical Fitness in 10 to 12 Years Old Boys. Phys. Act. Rev..

[53] Snijder M.B., van Dam R.M., Visser M., Seidell J.C. (2006). What Aspects of Body Fat Are Particularly Hazardous and How Do We Measure Them?. Int. J. Epidemiol..

[54] Mokdad A.H., Ford E.S., Bowman B.A., Dietz W.H., Vinicor F., Bales V.S., Marks J.S. (2003). Prevalence of Obesity, Diabetes, and Obesity-Related Health Risk Factors, 2001. J. Am. Med. Assoc..

[55] Villareal D.T., Apovian C.M., Kushner R.F., Klein S. (2005). Obesity in Older Adults: Technical Review and Position Statement of the American Society for Nutrition and NAASO, The Obesity Society. Am. J. Clin. Nutr..

[56] National Heart Lung and Blood Institute-the National Institutes of Health Assessing Your Weight and Health Risk. https://www.nhlbi.nih.gov/health/educational/lose_wt/risk.htm.

[57] Hinton P.S., Nigh P., Thyfault J. (2015). Effectiveness of Resistance Training or Jumping-Exercise to Increase Bone Mineral Density in Men with Low Bone Mass: A 12-Month Randomized, Clinical Trial. Bone.

[58] Kato T., Terashima T., Yamashita T., Hatanaka Y., Honda A., Umemura Y. (2006). Effect of Low-Repetition Jump Training on Bone Mineral Density in Young Women. J. Appl. Physiol..

